# Influence of trust on the acceptance of the RTS,S malaria vaccine in the Kassena-Nankana districts of Ghana

**DOI:** 10.1186/s12936-024-05180-x

**Published:** 2024-11-29

**Authors:** Eustace Bugase, Paulina Tindana

**Affiliations:** 1 University of the Witwatersrand, Johannesburg, South Africa; 2 School of Public Health, University of Ghana, P. O. Box 65 LG, Legon, Ghana

**Keywords:** Vaccines, Malaria, Trust, Acceptance

## Abstract

**Background:**

Vaccines have increasingly become some of the most effective public health tools for promoting health and reducing the burden of infectious diseases. The availability of a malaria vaccine for routine use will be a major milestone, nonetheless, trust by the public for the vaccine could pose a major challenge for its acceptance. Documented evidence such as the boycott of the oral polio vaccine in northern Nigeria and the failure of the Ebola vaccine trial in Ghana among others highlight the impact of public trust on vaccine acceptance.

**Methods:**

This is an explorative cross-sectional mixed-method study conducted in the Kassena Nankana districts. The study was conducted in the Kassena Nankana Districts between May and December 2020. A total of 390 structured questionnaires were administrated to mothers and caregivers of children under five years of age while 15 in-depth interviews were conducted with mothers and health workers. STATA software Version 16.0 was used to interpret the quantitative data, where bivariate and multivariate regression analysis was performed to determine the influence of trust on vaccine acceptance while QSR NVivo 12 software was used to code the qualitative data to aid the thematic analysis.

**Results:**

The results revealed that the level of knowledge of the RTS,S vaccine among participants was high. About 95.4% of the mothers had good knowledge of the malaria vaccine and more than half 61.2% of them got information about the vaccine from the health facility. The level of trust for the malaria vaccine was equally high with 91.4% of the mothers reporting that the vaccine treats childhood malaria. In a bivariate analysis, educational status (P = 0.013), ethnicity (P = 0.008), marital status (P = 0.041), education on the vaccine and perceived ineffectiveness P < 0.05, and trust for the malaria vaccine (P < 0.05) were found to be statistically associated with vaccine acceptance. Compared with participants who agree that vaccines are harmless to children, those who disagree were significantly less likely to accept vaccines (OR = 0.25, 95%CI [0.08, 0.83], p = 0.017). The qualitative data correspondingly revealed that mothers trusted vaccines which thus accounted for the high uptake of the malaria vaccine in the districts.

**Conclusion:**

The results of this study suggest that trust in the malaria vaccine is critical for its uptake. Therefore, efforts towards improving acceptance of the vaccine should be focused on building and sustaining trust for the vaccine among mothers and community members.

## Background

Vaccines are some of the most efficient public health tools for promoting health and reducing the burden of infectious diseases [1]. Recommendations for vaccines are constantly expanding, given the addition of new susceptible groups to the population that need immunization protection, and the development of greater evidence about the safety and benefits of vaccines [2]. While some children cannot be vaccinated for medical reasons, and in some areas, vaccines are not readily available, a growing number of children are not vaccinated or are vaccinated late due to their parents' conscious decision, often driven by misconceptions and mistrust [3]. A parent’s trust in the advice they receive from their child’s doctor correlates with confidence in vaccines, antibiotics, over-the-counter medications, and children’s vitamins [4].

Trust in vaccines and the health system is crucial for public health programmes that aim to deliver lifesaving vaccines [5]. Understanding the contributors and threats to trust is essential to explaining vaccine acceptance, particularly since they vary across epidemiological conditions, specific vaccines, and cultural and sociopolitical settings [5]. On the contrary, distrust in a healthcare system produces uncertainty and doubt, often leading to seeking second opinions or ‘alternative’ medical approaches [6]. Globally, more than 22 million children miss out on life saving vaccines every year, and every 20 seconds, a child dies from a vaccine-preventable disease [7].

Numerous examples show how public trust has influenced vaccine acceptance in developing countries. Public distrust led to the boycott of the oral polio vaccine in northern Nigeria in 2003 [8] and undermined polio eradication efforts in India [9] and the Democratic Republic of the Congo, where religious and traditional beliefs and distrust of government health services impeded vaccine programmes [7]. This evidence suggests that many vaccine boycotts and failures are not based on scientific evidence but rather incited by mistrust of vaccines.

The RTS,S/AS01 malaria vaccine is the first vaccine developed for any parasitic disease, marking a major milestone in the fight against malaria. Although its efficacy is modest, reducing malaria cases by 39% and severe malaria cases by 29% among children aged 5–17 months, it holds great potential to reduce child deaths in endemic areas [10]. The vaccine was introduced in Ghana in 2019 as part of a pilot programme. Ghana, alongside Kenya and Malawi, was selected due to its significant malaria burden, particularly in children under five[10]. The vaccine was administered in Ghana through the Expanded Programme on Immunization (EPI), follows a four-dose schedule for children aged six months to two years. The pilot was implemented in malaria endemic areas such as the Kassena Nanakana Municipality.

While the launch of the vaccine in Ghana was a significant step forward, it also sparked public concerns. Many people feared that the vaccine was a ploy to reduce the African race, generating mistrust [11]. Viral misinformation on social media, including WhatsApp, Facebook, and Twitter, fueled these misconceptions. The spread of such misinformation about a vaccine by the popular press could profoundly threaten the public’s trust for the vaccine and substantially affect its acceptability. It is, therefore, important to have a good understanding of the influence of trust on acceptance of vaccines, with the aim of providing substantial evidence to inform policy for the RTS,S malaria vaccine implementation to promote the comprehensive acceptance of the vaccine in the country.

## Aim of the study

This study aims to assess the influence of trust on the acceptance of the RTS,S malaria vaccine in the Kassena Nankana Municipality, Ghana. Understanding factors that influence vaccine acceptance is vital for designing effective health policies and client-centered interventions that improve vaccine uptake and support future vaccine deployment. Trust in vaccines is a key element in designing communication strategies that can measure progress toward achieving a malaria-free country. The findings from this study will offer relevant insights to the Ghana Health Service, international organizations, vaccine developers, and others working in public health to improve the acceptance and use of the malaria vaccine. Moreover, this study seeks to identify specific population segments that can be targeted with tailored information to combat misinformation and build community trust, ultimately contributing to more effective vaccination campaigns locally and nationally

## Methods

### Definition of study terms

#### Acceptance

Acceptance in this study is defined as the degree to which participants are willing to receive or endorse the use of vaccines, based on their attitudes, beliefs, and experiences.

#### Trust

In this study, "trust" is defined as the confidence participants have in the safety, efficacy, and delivery of vaccines, as well as in the healthcare system and providers administering the vaccines**.**

### Study design

**Fig. 1:**
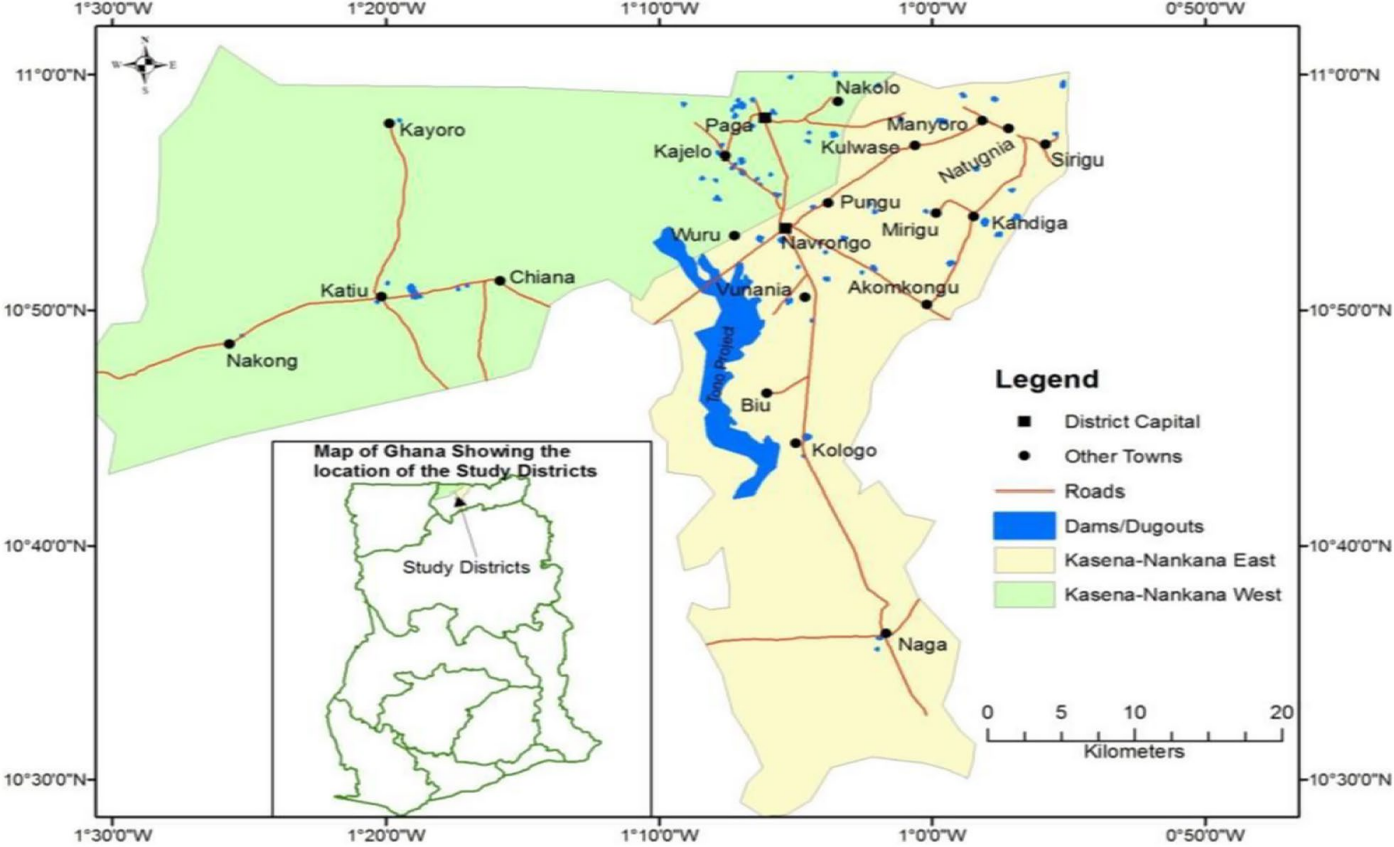
A map of Ghana showing the location of the study districts (Kassena-Nankana East and West Districts)

A cross-sectional study that employed both quantitative and qualitative techniques was used for the data collection. The study was conducted in the Kassena-Nankana East and West Districts of Northern Ghana from May to December 2020**,** coinciding with the introduction of the RTS,S malaria vaccine (Fig. 1). The quantitative survey presented figures and percentages on the research problem, the qualitative interviews were used to solicit in more detail the personal opinions and experiences of the people on the problem under investigation. The survey was conducted with mothers with children under five years old and qualitative interviews were conducted with both mothers and health workers within the study area. The study focuses on mothers because they are typically the primary decision-makers in matters of child health and vaccination in many cultural contexts, and thus, their perspectives are crucial in understanding vaccine acceptance and health behaviours.

### Description of data collection tools

The quantitative tool for this study was a structured questionnaire designed to assess mothers' knowledge, perceptions, and acceptance of the RTS,S malaria vaccine in the Kassena Nankana Municipality, Ghana. It included four main sections: (1) demographic data, (2) knowledge and perceptions of the RTS,S vaccine, (3) community factors influencing vaccine uptake, and (4) the influence of trust in vaccine acceptance. The questionnaire used closed-ended and a four-point Likert-scale questions scale ranging from "strongly agree" to "strongly disagree”. The questionnaire was developed based on existing literature and pre-tested for clarity and relevance before the study

For the qualitative interviews, a semi-structured interview guide was employed to explore caregivers' perspectives in depth. This guide consisted of open-ended questions designed to encourage participants to share detailed insights on their experiences with and perceptions of the RTS,S malaria vaccine. Probes were integrated into the guide to further explore key themes, such as perceptions of vaccine, the factors influencing their decision to accept or decline the vaccine, the role of trust in vaccine, healthcare providers and the broader healthcare system on vaccine acceptance.

### Study site

The study was conducted in the Kassena-Nankana East and west districts (KNEWDs) of northern Ghana. The area shares borders with Burkina Faso in the north and covers an area of about 1675 km2 and an estimated population of approximately 152000 under surveillance by the Navrongo health demographic surveillance system (NHDSS) operating under the NHRC [11] . The districts have two distinct seasons, a wet season that runs from May to September and a long dry season from October to April with hardly any rains. There are two main ethnic groups in the area, the Kasenas and the Nankani speaking people [12]. The community members are predominantly farmers. Malaria transmission in this part of Ghana is high with an entomological inoculation rate (EIR) of approximately between zero to 388 infective bites per person per year monthly [13].

Coverage of the EPI in the area is high: vaccination coverage for diptheria pertussis, tetanus, poliomyelitis, measles, yellow fever, and all basic immunization in the Kassena-Nankana districts of Ghana is over 95%. The districts have five health centres, two clinics, and 27 functional community-based health planning and services (CHPS) compounds located in various villages with resident community health officers (CHOs) offering doorstep health care services to the people. The districts have two district hospitals, and the main referral hospital (War Memorial Hospital) is located at the capital (Navrongo town) of the KNED.

The RTS,S/AS01 malaria vaccine (Mosquirix) was introduced in Ghana in April 2019 as part of a WHO-led pilot programme targeting children aged 5 to 18 months in regions with high malaria prevalence. In Ghana, the vaccine is available in selected areas, including the Northern, Upper West, and Upper East regions. In Kassena Nankana municipality, located in the Upper East Region, RTS,S is delivered through routine immunization services at health centres and clinics, with outreach programmes extending access to rural areas.

### Sample size calculation

The estimated sample size based on a population of 18,700 women with under-five children, obtained from the NHDSS unit of the NHRC. The Yamane (1997) formula for proportions was used to calculate the sample size. This calculation yielded an initial sample size of 390 mothers at a 5% margin of error and a 95% confidence level.

Formula;

$$n = \frac{N}{1 + N(e)2}$$

where,n= Sample Size N= Population size = Margin of error (0.05)n= 18700/1+ 18700*(0.05) (0.05)n= 390 participants

### Sampling technique

The Kassena-Nankana East and West Districts have a total of two hundred and twenty-two communities (220) which have been categorized into five (5) zones (Central, West, East, South, and West) by NHDSS [11]. By the GHS ethics committee standards for researching in the time of COVID pandemic, a face-to-face interview should be avoided as much as possible. Three (3) health facilities each were randomly selected from the five zones, resulting in a total of 15 selected facilities from which a contact list with the telephone numbers of all mothers was taken from each sampled facility for telephone interviews. The telephone numbers of mothers were then written out and selected at random from a basket. Participants were called and taken through the consent process and the questionnaire was administered to those who agreed to participate in the study.

For the qualitative interviews, purposive sampling was used to select study participants for the in- depth interviews. Participants were nurses/healthcare workers who were directly involved in administering the vaccine and knowledgeable mothers residing in communities within the five zones. Participants shared their experiences, opinions, and understanding of trust and vaccine acceptance in more detail, specifically to the malaria vaccine. These stakeholders were purposively selected because they possessed first-hand information and critical data on the RTS, S malaria vaccine deployment. Because of the small number of participants for the qualitative, they were interviewed face to face observing all COVID-19 protocols such as wearing of mask, observing social distancing, and using hand sanitizers.

### Data processing and analysis

The quantitative data was double-entered and verified using Epidata 4.0 with built-in consistency checks to control data input. Data cleaning by way of identifying outliers and checking consistencies among variables was carried out by running frequencies and cross-tabulations using STATA Version 16.0^©^. Descriptive statistics were used to describe the socio-demographic characteristics of respondents and to show patterns and trends of the questionnaire. The outcome of interest which is vaccine acceptance is considered as binomial. A bivariate analysis of background characteristics was conducted against the outcome variables. Chi-square (or Fisher’s Exact test) analysis was used to determine the relationship between factors that influenced trust and the outcome variable. A four-point Likert scale was used to measure the trust of mothers for vaccines. Bivariate and multivariate logistic regression was performed to assess the influence of trust on vaccine acceptance at a significance level of 95%.

All the qualitative interviews were audio-recorded and transcribed verbatim by trained transcribers, typed into MS word, and stored on a computer. Each of these transcripts was then critically reviewed by the researcher to ensure accuracy and to correct any errors before they were imported onto QSR Nvivo 12 (QSR International Pty Ltd, 2019) to facilitate data analysis. Guided by the objectives of the study and in relation to literature, a codebook was developed to guide the data analysis process. The results of the study were presented using quotes from the interviews to illuminate specific themes. The qualitative data was used to complement and clarify findings from the quantitative data.

## Results

### Socio-demographic characteristics of participants

Out of 390 mothers who took part in the survey, majority 230 (59%) of them were between the ages of 25 and 34 with the mean age being 27 years. Also, 31% (120) of the mothers had their education up to the secondary/senior High School level, 11.5% (45) never had formal education while about 15.1% (59) had tertiary/higher-level education. In terms of their marital status, majority of the respondents 74.1% (289) reported they were married while 9.5% (37) said they were never married with 2.5% (8) of them reporting that they were separated/divorced. We also found that 87.4% (341) of the mothers were Christians and 68.5% (267) of them were Kassenas. Out of the 390 mothers, 33.1% (129) were traders and 21% (82) were subsistence farmers. In terms of their formal employment status, 14.6% (57) were unemployed whereas 13.3% (52) were civil/public servants (Table 1)

**Table 1:** Socio-demographic characteristics of participants

Parameter	Count (N = 390)	Percentage
**Age category**		
16–20	34	8.7
21–34	314	89.2
35 +	42	10.8
**Educational level**		
No education	45	11.5
Primary/JSS	166	42.6
Secondary/Tertiary	120	30.8
Tertiary/ Higher	59	15.1
**Religion**		
Christian	341	87.4
Muslim	30	7.7
Traditional	19	4.9
**Ethnicity**		
Kassem	267	68.5
Nankam	109	28
Buli	14	3.5
**Marital status**		
Never married	37	9.5
Married	289	74.1
Living together	49	12.6
Separated/Divorce	8	2
Other	7	1.8
**Occupation**		
Farmer	82	21
Trader	129	33.1
Housewife	39	10
civil/public servant	52	13
unemployed	57	14.6

Source: Fieldwork, (2020)

### Bivariate analysis of the socio-demographic characteristics of mothers

Chi-square test (or Fisher Exact test as appropriate) of background characteristics revealed statistically significant relationship of educational status of mothers, marital status, and ethnicity with vaccine acceptance (P<0.05). The high level of education and the strong representation of married women in this population potentially contributed to the high trust in the RTS,S malaria vaccine observed in this study (Table 2)

**Table 2:** Socio-demographic characteristic and vaccine acceptance

Factors	Vaccine uptake		P-value
*Age group*	Acceptance n (%)	Non-acceptance n (%)	
16–20	6 (11.11)	28 (8.33)	0.168
21–34	46 (85.19)	268 (79.76)	
35 +	2 (3.70)	40 (11.90)	
*Educational level*			
No education	5 (9.26)	40 (11.90)	0.013
Primary/JSS	26 (48.15)	140 (41.67)	
Secondary +	23 (42.59)	156 (46.43)	
*Religion*			
Christian	50 (92.59)	17 (5.06)	0.528 ^F^
Traditional	2 (3.70)	291 (86.61)	
Muslim	2 (3.70)	28 (8.33)	
*Ethnicity*			*0.037 ^F^
Kassem	35 (64.81)	232 (69.05)	
Nankam	18 (33.33)	91 (27.08)	
*Marital status*			*0.015 ^F^
Never married	9 (16.67)	28 (8.33)	
Married	36 (66.67)	253 (75.30)	
Living together	7 (12.96)	42 (12.50)	
Separated/Divorce	2 (3.70)	6 (1.79)	
Other	0 (0.00)	7 (2.08)	
*Occupation*			0.303
Farmer	12 (22.22)	70 (20.83)	
Trader	18 (33.33)	111 (33.04)	
Housewife	7 (12.96)	32 (9.52)	
Civil/public servant	7 (12.96)	45 (13.39)	
unemployed	5 (9.26)	52 (15.48)	
Others	5 (9.26)	26 (7.74)	

^F^ = Fisher’s exact test; *p < 0.05

Source: Fieldwork, (2020)

Mothers aged 35 and above were more likely to accept vaccines compared to those aged 20 and below, possibly due to the greater experience older mothers have with vaccines. However, this association was not statistically significant. The results also showed that married mothers were significantly more likely to accept vaccines than those who were never married, which could be attributed to the support and encouragement they receive from their spouses and other relatives.

### Mothers’ knowledge of vaccines

Knowledge of vaccines in this study was found to be high among participants. From the results, about 86.4% (337) of the mothers supported vaccines to be given to children. Also, the results showed that 95.6% (372) of the mothers were aware of the malaria RTS, S vaccine, and more than half 62.3% (243) reported hearing of the vaccine from the health worker during ANC visits. Majority 61.7% (241) of the participants reported they heard the vaccine was good. Over-all, majority 95.6% (373) of the participants had good knowledge of the vaccine. About 92.8% of the mothers who had good knowledge about the vaccine indicated they will accept the vaccine. Good knowledge in this study meant mothers have heard of the vaccines and are aware of its true purpose of preventing malaria in children.

In a bivariate analysis model, we found that the likelihood of accepting the RTS, S malaria vaccine was approximately 4.6 times higher among mothers with good knowledge as compared to mothers with poor knowledge of the vaccines. This association was statistically significant (P<0.001). This knowledge served as a foundation for building trust in the RTS,S vaccine, as evidenced by the strong association between mothers’ trust in the vaccine and their decision to vaccinate their children (Table 3)

**Table 3:** Bivariate analysis of knowledge factors that influence vaccine acceptance

Variables	Vaccine acceptance		Total	χ^2^ (p- value)	COR (95%CI) p-value
	Non-acceptance	Acceptance			
	n (%)	n (%)			
Knowledge level				(0.000)^F^*	
Poor knowledge	15 (88.2)	2 (11.8)	17 (100)		1
Good knowledge	27 (7.2)	346 (92.8)	373 (100)		4.61 (2.8, 7.4) 0.001

Source: Field data, (2020)

Respondents in the qualitative interviews expressed similar views. Most of the mothers and health workers exhibited good knowledge of vaccines in general and the RTS,S malaria vaccine in particular. They identified the malaria vaccine in the local language as *‘Paa gariloi’* and ‘*malaria Paniea’* (to wit malaria injection) for both the Kassenas and Nankanas, respectively. Participants expressed a positive outlook on the malaria vaccine and regarded it as a good measure of keeping their children from malaria infection. The following expressions from the participants echo the knowledge of vaccines among mothers.

***Q:**** Have you heard about the RTS,S malaria vaccine and what did you hear?*

***R:**** “Yes, I learned it is given to protect children who are less than five years from getting malaria and the child is to take it four times, this is what I was told at the Health Centre during ANC and PNC visits.” (****IDI-30year old mother****)*

***R: “****According to the nurses, that vaccine protects our children from contracting malaria. They said, we use mosquito nets but still malaria is there, and I learned that when under- five children get malaria, some die and some also get complications so they introduced the vaccine to protect our children from getting malaria and the child takes it four times. This was what I was told when I went for weighing****” (IDI-35year old mother)***

Similarly, good knowledge of the vaccine was exhibited by the health workers. They had in-depth knowledge about the vaccine and stated they received the information from workshops they attended before the introduction of the vaccine. The following expression from the health workers resonate the knowledge of the vaccine among them.

***R: “****What I know of the RTS,S is that it is given to children aged 6 months to 2years and is given four times in one’s life. The first one is given 6 months, the second 7 months skip the* *8*^*th*^*-month wait 9 months you give and if the child turns 2years it is given to them. This vaccine prevents children from getting malaria. Also, we have side effects that are something like headache, fever, rashes, diarrhea just to mention a few. In summary, that is what I know about the vaccine****.” (IDI-Nurse, Navrongo).***

### Association of factors that influence vaccine acceptance

The results highlighted the significant influence of trust on vaccine acceptance. Mothers who expressed trust in the effectiveness of the RTS,S malaria vaccine were significantly more likely to accept the vaccine for their children. In contrast, mothers who doubted the vaccine’s effectiveness were less likely to vaccinate, emphasizing the critical role of trust in shaping vaccine behavior as compared to mothers who perceive the vaccine to be effective.

The results indicated that mothers who cited community involvement as a factor influencing their decision to accept vaccines were more likely to do so compared to those who did not, although this association was not statistically significant. Additionally, mothers who considered the vaccination process time-consuming had approximately 4.1 times higher odds of accepting the vaccine compared to those who did not, but this association was also not statistically significant (Table 4). Furthermore, mothers who identified an inadequately informed consent process as a factor were 1.22 times more likely to accept vaccines than those who did not, though this relationship was not statistically significant.

**Table 4:** Bivariate analysis of factors that affect vaccine acceptance

Factors	Vaccine acceptance		χ^2^ (p-value)	COR (95%CI) p-value
	Non acceptance	Acceptance		
	N (%)	N (%)		
Perceived ineffectiveness of vaccine	16(38.10)79(22.70)		4.82 (0.028)*	0.48 (0.24, 0.93) 0.031 *
Adequate education	28 (66.67)229(65.9)		0.01 (0.911)	0.96 (0.49, 1.90) 0.911
Lack of community involvem’t	10 (23.81)97 (27.87)		0.31 (0.577)	1.24 (0.59, 2.61) 0.578
Lack of ethical guidelines	8 (19.05)43 (12.36)		1.48(0.224)	0.60 (0.26, 1.38) 0.229
Risk of using new drugs	16 (38.10)127 (36.49)		0.04(0.839)	0.93 (0.48, 1.81) 0.839
Time-consuming	1 (2.38)31 (8.91)		(0.231)^F^	4.01 (0.53, 30.16) 0.177
Inadequate informed consent procedure	2 (4.76)20 (5.75)		(1.000) ^F^	1.22 (0.27, 5.41) 0.794

F = Fisher’s exact test

Source: Fieldwork, (2020)

The qualitative interviews with the mothers recounted health education about vaccines and perceived benefits of vaccines as factors that influenced their decision to accept vaccines.

Additionally, the mothers reported that the previous experience and benefits from some vaccines contributed to their positive perception they have about the effectiveness of the RTS,S vaccine. Excerpts from the mothers explained this;

***Q****. What are the factors that influence your decision to accept vaccines?*

***R.*** “*Well that was not my first child, the previous ones that took vaccines are doing well and not falling sick, so I see vaccines to be effective in preventing diseases and this is the reason why when I give birth, I allow my children to be vaccinated****.” (IDI-32-year-old mother)***

***R. “****It is the education given by the nurses during weighing (ANC). Also, during home visits, they educate we the nursing mothers on the importance and benefits of immunization to the child. And for every vaccine the child is taking we are told the benefits to the child. Above all when our children take the vaccine, they do not easily fall sick like that and you will see that the vaccine is effective and the child grows well and they are healthy and all that****.” (IDI-35year old mother)***

The health workers expressed similar views as the mothers. They emphasized the relevance of giving the right information and education about vaccines to the mothers. They also indicated previous experience with vaccines as well as the perceived effectiveness to be some other factors that influence the acceptance of vaccines. The narratives from the nurses buttress this claim.

***R****. “I think the effectiveness of the vaccine and mothers' own experiences with vaccines is what influences them. Most times after having your first child and experiencing good health the child enjoys the mother's build some trust, and so they believe the drugs are good and so, should continue to take them. I think it is just from their personal experiences and the*

*education they receive. When there is a new vaccine to be introduced, there is a lot of education, community engagement ,here and there, before the vaccine is being introduced so they get to hear and learn a lot from that, and their personal experiences too counts****.” (IDI-Nurse, Chiana )***

***R**** “It is because they see that the vaccine is effective from looking at previous years of experience with vaccines. Let’s consider a vaccine like measles, it was clearing every child. At that time if you had about 10 children, you knew that you still did not have children yet. But this time, they have seen that once they have taken the vaccines, they were not seeing such deaths again, so most of them saw that the vaccine was working. That is why they are coming. They have seen that the vaccine is productive and yields benefits.” ****(IDI- Nurse, central)***

### Trust and vaccine acceptance

Trust in this study was considered a binomial variable. Trust was assessed using a four-point Likert scale (strongly agree, agree, strongly disagree, and disagree). Participants agreed or disagreed with statements to indicate their level of trust.

From the results, 95.4% (372) of the mothers trusted vaccines to be good, whiles 94.4 % (368) of the mothers’ trust vaccination to be the most efficient way to prevent disease of children under five years of age. The data showed that 91.5% (357) of the mother agree with the statement that the RTS,S malaria vaccine can not cause any harm to their children and 95. 4% of the mothers trusted vaccines. Also, 94.4% of the participants trusted childhood vaccinations, while 95.6% trusted the health worker .The results revealed that mothers' trust in, childhood vaccination, the health worker, and trust in the malaria vaccine were found to be associated with vaccine acceptance, but not statistically significant.

The results confirmed that trust in the RTS,S malaria vaccine was the primary driver of vaccine acceptance. Mothers who believed that vaccines are harmless were significantly more likely to accept the RTS,S vaccine for their children (OR = 0.25, 95% CI [0.08, 0.83], P = 0.017). Qualitative data also revealed that trust in the vaccine, bolstered by positive experiences with previous vaccines and reassurance from healthcare workers, was a critical factor in mothers’ decisions to vaccinate their children (Table 5).

**Table 5:** Association of trust perceptions with vaccine acceptance

	Coefficient (β)	Crude Odds Ratio	95%CI	P-value
**Predictor factor**				
***Vaccines are harmless***				
Agree		1.00		
Disagree	1.373	0.25	[0.08, 0.83]	0.017
***Vaccine prevents children less 5 yr from being sick***				
Agree		1.00		
Disagree	0.040	0.96	[0.29, 4.01]	0.951
***Health workers administering childhood vaccines are qualified***				
Agree		1.00		
Disagree	1.16	3.20	[0.59, 60.13]	0.275
***Health workers will treat adverse event promptly***				
Agree		1.00		
Disagree	0.277	0.76	[0.41, 1.46]	0.391
***Health workers are friendly***				
Agree		1.00		
Disagree	0.219	1.34	[0.32, 4.82]	0.702

Source: Field data, (2020)

Correspondingly in the qualitative interviews, the views expressed by mothers suggested that trust in vaccines played an important role in their decision to allow their children to be vaccinated. Many of them reported that the education given about the vaccine during ANC built their trust for the vaccine. The mothers expressed their views on the issue this way:

***Q****: Has your child taken the malaria vaccine and why?*

***R****: Yes, my baby has taken it. I have trust in the vaccine that it will help my baby and keep her healthy. I told you; I know the vaccine is to prevent the child from getting malaria, besides, that particular injection is good for them and that is my only reason. (****IDI with 29year old mother****)*

Likewise, from the health workers, trust in the vaccines is the main reason mothers come for the vaccines. They emphasized this stating that, the trust for the vaccine originated from the numerous benefits gotten from previous vaccines. Therefore, the belief the mothers have in the malaria vaccines was the main predictor. The narratives by the nurses explain this;

*Q. Do you think trust plays a role in mother's acceptance of the malaria vaccine?*

*R. “Well, I think trust does play a crucial role and the trust I think is much in the vaccine because if their trust were in the nurse, this is my first month here, and they do not know me, and yet they still bring their children for vaccinations. And so, it is not trusting in the nurse that motivates them to come for vaccination other than that since we came as new nurses, the mothers wouldn’t have come. They’ll wait till they gain that trust in us before they come. They trust in the vaccine, and it is because the vaccines work to give the immunity the children need. that’s why they come but not because of any person.” (****IDI- Nurse, Chiana)***

## Discussion

### Main findings of the study

This study found that knowledge of the RTSS malaria vaccine was widespread among mothers in the district, with antenatal clinics (ANC) serving as the primary source of information. Mothers with good knowledge of the vaccine were significantly more likely to accept it (OR=4.61, 95% CI [2.8, 7.4]). Educational status emerged as a significant factor influencing vaccine acceptance, as formally educated mothers demonstrated higher levels of acceptance. Although marital status was not statistically significant, married mothers exhibited greater odds of accepting the vaccine compared to unmarried mothers. Ethnicity was another key factor, with Kasem mothers more likely to accept the vaccine than Nankani mothers. Additionally, perceived vaccine effectiveness and trust were critical in driving vaccine acceptance, with trust being particularly shaped by the district’s history of successful vaccine trials conducted by the NHRC. Compared with participants who agree that vaccines are harmless to children, those who disagree were significantly less likely to accept vaccines (OR =0.25, 95%CI [0.08, 0.83], p = 0.017)

### Implications and generalizability

Our findings underscore the importance of ANC as a vital platform for educating mothers about vaccines. In the study area, strengthening ANC-based education on vaccines could improve uptake rates, especially among less educated and ethnically diverse groups. This approach is relevant beyond the district, as it aligns with broader national health strategies in Ghana and could be effective in other rural areas where access to health information is limited. On a global scale, these results support the use of routine maternal health services to disseminate vaccine information, particularly in low- and middle-income countries. The association between knowledge, education, trust, and vaccine acceptance highlights the need for targeted communication strategies that account for local cultural and educational contexts.

### Comparison with other literature

#### Knowledge of vaccine

Knowledge of vaccines was widespread among mothers in the study area. This finding is analogous to the findings of a study in Kenya [14]. The results indicated that majority of the mothers had knowledge of the RTSS malaria vaccine from various sources. Profoundly, most of the mothers representing 62.3% identified ANC clinic as their main sources of information concerning the vaccine. This finding was affirmed by a study that indicated that 63% of mothers got information on routine vaccination at the antenatal clinics (ANC) [15]. Also, showing from the results, almost all of the mothers that got their information from the health worker during ANC visits had comprehensive knowledge about the vaccine which had a positive implication on the acceptability of the vaccine. Therefore, suggesting from this finding, health care workers should endeavour to educate mothers about vaccines to help curb the spread of misinformation and bad rumours about vaccines among mothers. Also, mothers should be encouraged to attend ANC regularly so they do not miss out on valuable information concerning vaccines.

Good knowledge in this study was found to be significantly associated with vaccine acceptance (OR=4.61 95% [2.8, 7.4]). Mothers who had good knowledge of the malaria vaccine were 4.6 times more likely to have their children vaccinated as compared to mothers who had poor knowledge. Our finding corresponds with earlier results of studies that reported mother's knowledge about vaccination as a predictor of full vaccination of their children [15, 16]. The widespread knowledge of vaccines among the mothers in the districts could be due to the long history of clinical, and vaccine trials conducted by the NHRC in the communities.

### Factors that influenced mothers’ acceptance of vaccines

The results indicated that the educational status of the mother was associated with vaccine acceptance. Mothers with formal education were more likely to have a good understanding and make decisive decisions on vaccinating their babies as compared to the uneducated mothers. The trust instilled in educated mothers may stem from their enhanced ability to access and interpret information, further supporting the critical role that trust plays in vaccine acceptance. Based on the data gathered, this study could perhaps conclude that misinformation and noncompliance to vaccination of babies could be erased if mothers had an education. Our finding concurs with an outcome of a study that reported the educational status of mothers as a determinant of their uptake of vaccines for their babies [17].

Additionally, marital status in this study was found to be associated with the uptake of vaccines, although the relationship was not statistically significant. The odds of vaccine acceptance were approximately 2.6 times among married mothers as compared to those not married. This could be due to the support, encouragement, and reminders given by their spouses and family members concerning vaccinations. However, some of the subgroups analysed, such as mothers who were never married, had relatively small sample sizes. This may limit the statistical power of the bivariate analyses and the generalizability of the findings. Therefore, the findings are interpreted with caution. This discovery is confirmed by the results of a study where the marital status of mothers was associated with uptake of childhood vaccination [18].

Also, the finding from this study indicates that ethnicity played a role in the acceptance of vaccines. According to our findings, there were higher chances of a Kasem mother accepting vaccines as compared to a Nankam mother, which are the two main tribes in the district. This outcome corresponds with the conclusion from the measles vaccine and human papillomavirus, awareness and acceptability study, where ethnicity was found to be associated with vaccine awareness and acceptance [19]. This observation suggests therefore that people’s values, preferences, and beliefs could somehow constrain their acceptance of a particular health care intervention.

Furthermore, our study found some intermediary factors that were associated with mothers' acceptance of vaccines. Our findings revealed that perceived vaccine effectiveness and education about the vaccine played a role in shaping trust. Mothers who reported the malaria vaccine to be effective were more likely to have their children vaccinated as compared to mothers who perceived the vaccine to be ineffective. The benefits of vaccines and the positive experiences mothers have with vaccination in the district account for this finding. Furthermore, this finding concurs with that of the finding from a qualitative study where the final acceptance of new health strategies was reported to be influenced by the perceived general effectiveness of the intervention [20]. This finding concurs with that of a qualitative study on factors likely to affect community acceptance of a malaria vaccine in Ghana, where the final acceptance of new health strategies was reported to be influenced by the perceived general effectiveness of the intervention [21].

### Trust on vaccine acceptance

Our study found that the mother's trust in vaccines was responsible for compliance to vaccinations in the communities. The analysis indicates that trust, particularly the belief that vaccines are harmless, is associated with higher vaccine acceptance, However, it is essential to approach these findings cautiously, as trust is just one of many factors impacting mothers' decisions. We acknowledge that trust is an important determinant of vaccine uptake, but it is not the sole factor influencing mothers' decisions. Therefore, while trust is a critical component of public health strategies, it should be viewed as part of a broader framework that includes multiple factors affecting vaccine behavior. Ostensibly, this trust likely stems from the positive outcomes of previous vaccinations and the strong relationships fostered by the Navrongo Health Research Centre in conducting research and vaccine trials in the districts over the years.

This finding corresponds to that of a qualitative analysis of mothers’ decision-making about vaccines for infants study where lack of trust was reported as a central factor in mothers’ decisions to vaccinate their children [22]. Inferring from this finding, it is substantial to provide information about vaccines and also to maintain a good relationship with mothers in other to build and sustain trust with the mothers who are concerned with vaccinating their children.

Additionally, a study conducted in Kenya aligns with the findings that trust plays a pivotal role in vaccine uptake. The study found that caregivers' trust in the RTS,S malaria vaccine increased over time, particularly as concerns about side effects diminished and the perceived benefits of the vaccine, such as reduced malaria episodes, became evident[23]. Similar to our findings, the authors caution that trust alone is insufficient to guarantee vaccine uptake. Systemic barriers, including negative provider attitudes, vaccine stockouts, and logistical challenges, were equally significant in determining whether caregivers completed the vaccination schedule. Thus, while trust in vaccines is a crucial factor, as highlighted in both studies, comprehensive public health strategies must also address healthcare system constraints and improve provider-patient interactions to enhance vaccine uptake.

## Future research and priorities

Moving forward, future studies should focus on interventions that enhance ANC-based education and expand outreach to mothers with lower education levels and those from different ethnic backgrounds. Qualitative research could provide deeper insights into trust, the cultural and ethnic factors that influence vaccine acceptance. As new vaccines, such as booster doses for malaria or vaccines for other infectious diseases like dengue or Ebola, become available, it will be important to examine how the factors of trust, knowledge, and perceived effectiveness interact with the introduction of these vaccines. In the context of malaria, improving vaccine uptake through education and trust-building is critical for the success of the RTS,S vaccine and future malaria control efforts.

## Limitations of the study

Due to the COVID-19 pandemic and the resulting restrictions, including lockdowns, social distancing measures, and other protocols, physical contact with participants was limited. This constraint hindered the ability to collect in-depth data for the qualitative interviews with the mothers, which significantly impacted the qualitative component of the study.

One key limitation of this study is the use of phone-based surveys, which restricted participation to mothers with access to a phone and a stable connection. This may have introduced selection bias, as mothers without access were excluded, potentially affecting the representativeness of the sample. This factor limits the generalizability of the results to the broader population.

## Conclusion

With the surging effort to eliminate vaccine-preventable disease and reduce under-five mortality, it is essential to highlight the crucial role trust plays in the uptake of vaccines. Whereas many studies in the Kassena-Nankana districts have looked at community factors relating to vaccine acceptance, not much is known of the specific role trust plays at the community level on vaccine acceptance. Compliance with existing vaccines is high in the study area due to the positive impact of vaccines on the health status of people particularly children under five years.

This study emphasizes the critical role of trust in vaccine acceptance. While factors such as education and knowledge about the vaccine were important, it was the trust that mothers placed in the RTS,S malaria vaccine that most significantly influenced their decision to vaccinate their children. Public health campaigns should focus on building and sustaining trust through transparent communication and ongoing engagement with communities to ensure the successful deployment of future vaccines.

## Data Availability

No datasets were generated or analysed during the current study.
